# Data on the generation of two *Nr2e3* mouse models by CRISPR / Cas9D10A nickase

**DOI:** 10.1016/j.dib.2020.106447

**Published:** 2020-10-21

**Authors:** Izarbe Aísa-Marín, M. José López-Iniesta, Gemma Marfany

**Affiliations:** a Department of Genetics, Microbiology and Statistics, Universitat de Barcelona, Avda. Diagonal 643, Barcelona 08028, Spain; b CIBERER, ISCIII, Universitat de Barcelona, Barcelona, Spain; c Institute of Biomedicine (IBUB, IBUB-IRSJD), Universitat de Barcelona, Barcelona, Spain; d MaRCU - Molecular and RNA Cancer Unit, Graduate School of Medicine, Kyoto University, Kyoto, Japan

**Keywords:** *Nr2e3*, CRISPR, Cas9 D10A nickase, Mouse models, Inherited retinal dystrophies, Retinitis pigmentosa, Enhanced S-cone syndrome

## Abstract

*NR2E3* encodes an orphan nuclear receptor that plays a dual function as both transcriptional activator and repressor in photoreceptors, being necessary for cone fate inhibition as well as rod differentiation and homeostasis. Mutations in this gene cause retinitis pigmentosa (RP), enhanced S cone syndrome (ESCS) and Goldmann-Favre syndrome (GFS). There is one reported *Nr2e3* isoform that contains all 8 exons and a second –previously unreported– shorter isoform, which only spans the first 7 exons and whose function is still unknown. In this data article, we designed and generated two new mouse models by targeting exon 8 of *Nr2e3* using the CRISPR/Cas9-D10A nickase in order to dissect the role of the two isoforms in *Nr2e3* function and elucidate the different disease mechanisms caused by *NR2E3* mutations. This strategy generated several modified alleles that altered the coding sequence of the last exon thereby affecting functional domains of the transcription factor. Allele Δ27 is an in-frame deletion of 27 bp that ablates the dimerization domain, whereas allele ΔE8 (full deletion of exon 8), produces only the short isoform that lacks the dimerization and repressor domains. Morphological and functional alterations of both Δ27 and ΔE8 mutants are reported in the associated research article “*Nr2e3* functional domain ablation by CRISPR-Cas9D10A identifies a new isoform and generated Retinitis Pigmentosa and Enhanced S-cone Syndrome models” (Aísa-Marín et al., 2020).

**Specifications Table**

**Table:** 

Subject	Genetics
Specific subject area	Generation of two *Nr2e3* mouse models by CRISPR/Cas9-D10A
Type of data	Table, Figure
How data were acquired	Generation of a murine model by gene editing in the zygote, genomic PCR, Sanger DNA sequencing.
Data format	Raw, analyzed
Parameters for data collection	*Nr2e3* mutant compared to wild-type mice.
Description of data collection	One cell/First-stage embryos were microinjected with RNAs coding for guide RNAs and Cas9D10A nickase. Products of specifically designed PCRs on mosaic founder gene-edited mice were electrophoresed to detect the generation of different alleles. Sanger sequencing of the CRISPR-deleted alleles was performed to validate targeting and resulting deletions.
Data source location	Universitat de Barcelona. Barcelona, Spain. Latitude and longitude (and GPS coordinates) for collected samples/data: 41.385634 ° N, 2.120092 ° E
Data accessibility	Within this Data in Brief article.
Related research article	Aísa-Marín, I. López-Iniesta, M.J. Milla, S. Lillo, J. Navarro, G. de la Villa, P. Marfany, G. *Nr2e3* functional domain ablation by CRISPR-Cas9D10A identifies a new isoform and generates Retinitis Pigmentosa and Enhanced S-cone Syndrome models. *Neurobiol. Dis.***146**, 2020, 105122.

## Value of the Data

• The presented data give a detailed account of the generation of two mouse models caused by mutations in exon 8 of *Nr2e3* using the CRISPR/Cas9 system. The Cas9-D10A nickase (requiring the use of 4 different guide RNAS) was used to prevent potential off-target effects.
• These data may provide useful information for researchers who seek better understanding of CRISPR/Cas9-D10A modifications (using the nickase mutant enzyme) and the generation of new mouse models, as well as researchers interested in using the novel ∆27 and the ∆E8 *Nr2e3* mouse models because of their resemblance to human-related phenotypes.
• These data may stimulate further investigations comparing the molecular mechanisms of the two diseases caused by mutations in *NR2E3*, Retinitis Pigmentosa and Enhanced S-cone Syndrome, in humans and mice. The novel mice models may also provide an instrumental tool for evaluating disease progression and therapeutic efficacy since the ∆E8 mutant is the first model of Retinitis Pigmentosa caused by mutations in *Nr2e3*. From the technical point of view, other researchers may be interested in comparing methods for minimizing potential off-target effects of the full Cas9, or in the efficiency of the Cas9 D10A nickase for the generation of medium-large size deletions.

## 1 Data Description

In this data article, we first designed the deletion of exon 8 of *Nr2e3* using the CRISPR-Cas9 system. The CRISPR-Cas9 system, which is widely used to target genes and generate modifications in the genome, has two components: a *cis* proto-space adjacent motif (PAM) next to the target site, and a *trans* guide RNA (gRNA) complementary to the target DNA. gRNAs are normally designed to be specific for the desired target site, however, in large genomes such as those of rodents, there are often similar sequences with a few mismatches that may be recognized by Cas9, thereby representing potential off-target effects. Actually, off-target effects are the main concern of the CRISPR-Cas9 system usage, as it may negatively affect experimental results, especially in clinical applications [2], [3], [4], [5], [6], [7], [8], [9], [10], [11], [12]. We thus explored the use of the mutant variant Cas9 D10A, which produces nicks on one strand of the DNA. In this case and to induce one double strand break (DSB), two gRNAs closely located in the genome target site (each with its PAM sequence motif nearby) have to be designed. If the gene-editing design intends a medium/large size deletion, then four different gRNAs have to be designed (two per each flanking DSB), which strongly minimize the probability of undesired off-target site cuts [13], [14], [15], [16], [17].

**Fig. 1:**
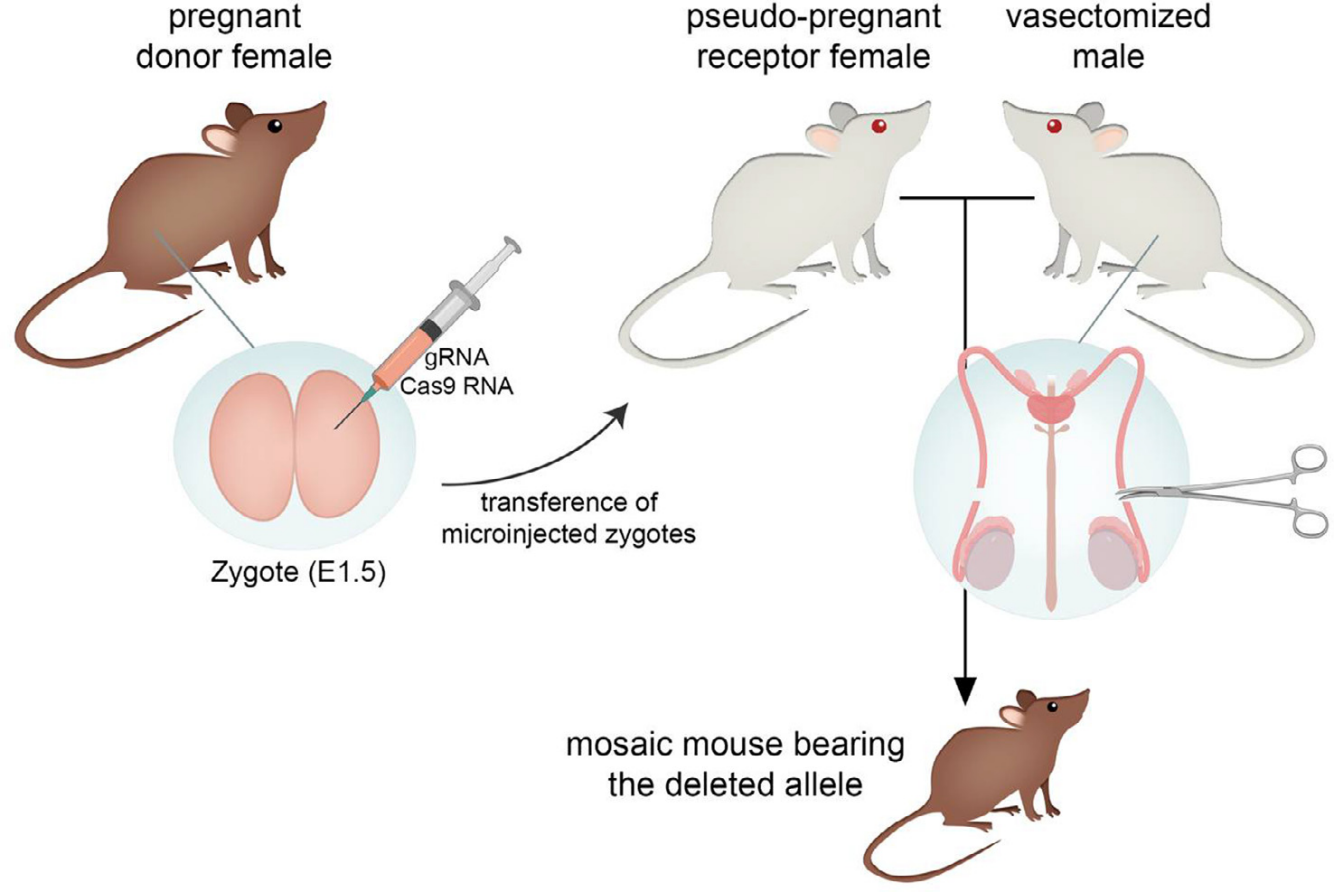
**Generation of gene-edited mice using the CRISPR/Cas9-D10A system.** Embryos from pregnant donor females were extracted in a one- or two-cell state and were microinjected with the four gRNAs and the Cas8-D10A RNA. Receptor females were mated with vasectomized males, which present an escision of the testis vas deferens, to achieve the state of pseudo-pregnancy. The modified embryos were transferred to the pseudo-pregnant receptor females, thus obtaining mosaic mice bearing different deleted alleles.

**Fig. 2:**
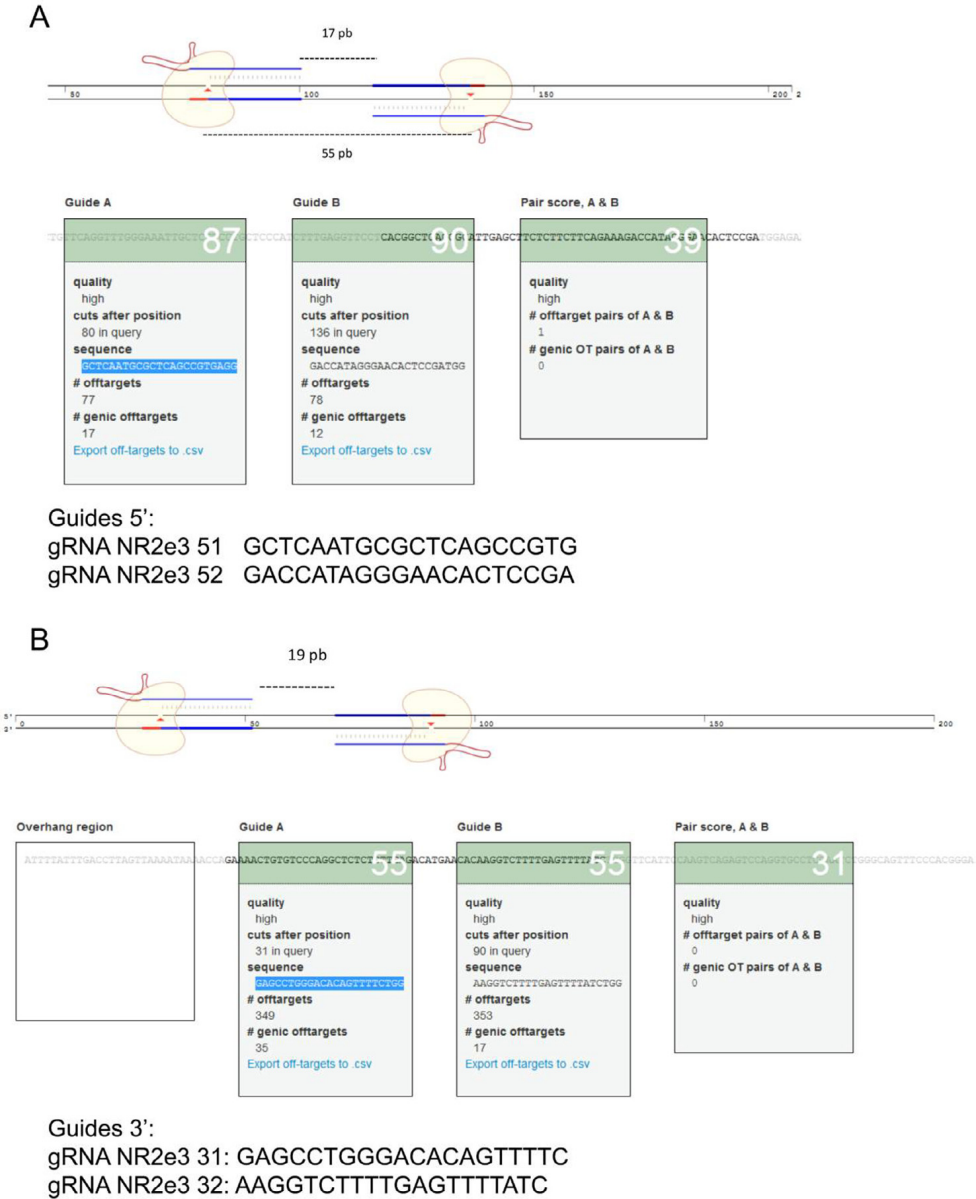
**CRISPR/Cas9–10A guide position.** To perform *Nr2e3* gene edition with the Cas9-D10A nickase, we used four guides, **(A)** two guides at 5′, and **(B)** two guides at 3′. Selected guide sequences and position are depicted.

**Fig. 3:**
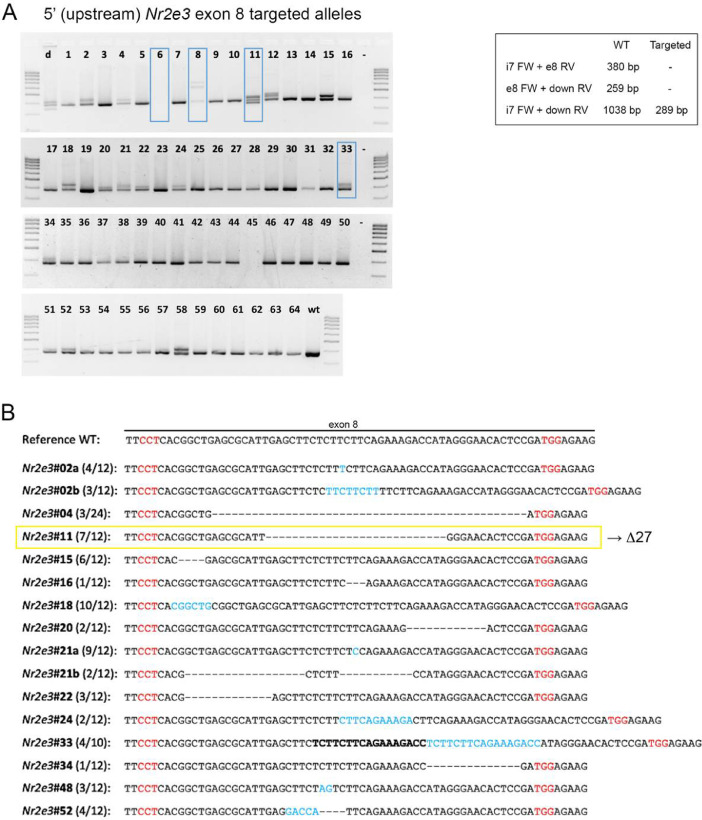
**Generation of *Nr2e3* mutant alleles modified at 5′ of exon 8. A)** Genotyping of gene-edited alleles at the 5′ position (junction of intron 7 and exon 8) showing the products of highly specific and discriminative PCR tests. The amplification of additional PCR bands and heteroduplexes indicated mosaic mice carrying different gene-edited alleles. Blue boxes indicate mice selected for further analysis. Lane 11 corresponds to the mosaic mouse carrying the Δ27 mutant allele. The predicted size of the PCR products for the wildtype and targeted mutant alleles is indicated within the box. **C)** Sequence of PCR bands amplified from gene-edited animals showing different *Nr2e3* alleles solely edited at the 5′-end. Many modified alleles with partial deletions and modifications at the junction of intron7- exon8 were generated. In red, PAM sequences; in blue, additional nucleotides added after DNA repair of the DSB. Dashes indicate deleted sequences, whereas the sequence in bold indicates a duplicated sequence in tandem.

**Fig. 4:**
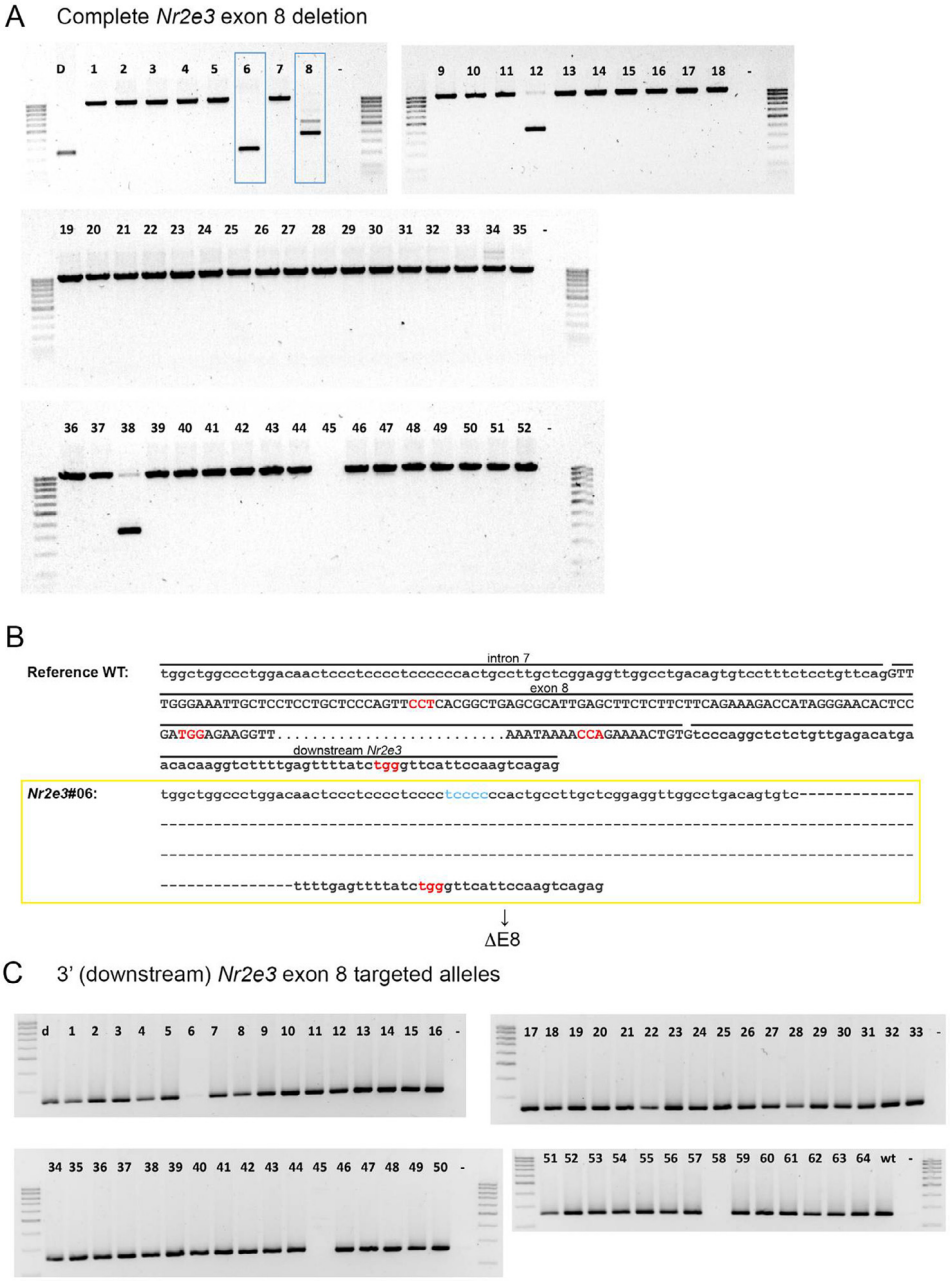
**Generation of *Nr2e3* mutant alleles containing the whole deletion of exon 8. A)** Genotyping of gene-edited alleles for full exon 8 deletion, showing the resulting specific PCR products. Blue boxes indicate interesting modified alleles. Lane 6 corresponds with the ΔE8 mutant. **B)** Sequence of the PCR band amplified from gene-edited animals with the complete targeted deletion. Only 1 of the 64 microinjected mice carried the complete exon 8 deletion. In red, PAM sequences; in blue, additional nucleotides added after DNA repair of the DSB. Dashes indicate deleted sequences, whereas the sequence in bold indicates a duplicated sequence in tandem. **C)** Genotyping of gene-edited alleles at the 3′ position showing the PCR products.

To generate the genetically modified mice, embryos from pregnant donor females were extracted, manipulated in vitro and then transferred to pseudo-pregnant receptor females (Fig. 1). As aforementioned, we opted for the use of the D10A Cas9 nickase and therefore, we used a total of 4 guide RNAs to delete exon 8 (guide design and position in Fig. 2). These four gRNAs were microinjected together with the mRNA encoding the Cas9D10A nickase into in vitro fertilized zygotes. In order to identify potentially modified alleles, we first used the T7 endonuclease assay, but immediately designed a highly specific and discriminative PCR test (specific primers and electrophoresis gels with high percentage agarose, 3%), which allowed the detection of different size alleles and heteroduplexes. The presence of gene-edited alleles in mosaic mice (shown in Figs. 3 and 4) were detected by PCR and confirmed by Sanger sequencing. Most of the gene-edited alleles were only modified at the junction of intron 7 with exon 8 (Fig. 3**A and** 3**B**). Only one allele was modified containing the whole deletion (Fig. 4**A and** 4**B**) and no modifications were found only affecting in the 3′UTR region (Fig. 4**C**), which strongly suggest that the 4 gRNAs were not equally efficient in targeting their sites.

**Fig. 5:**
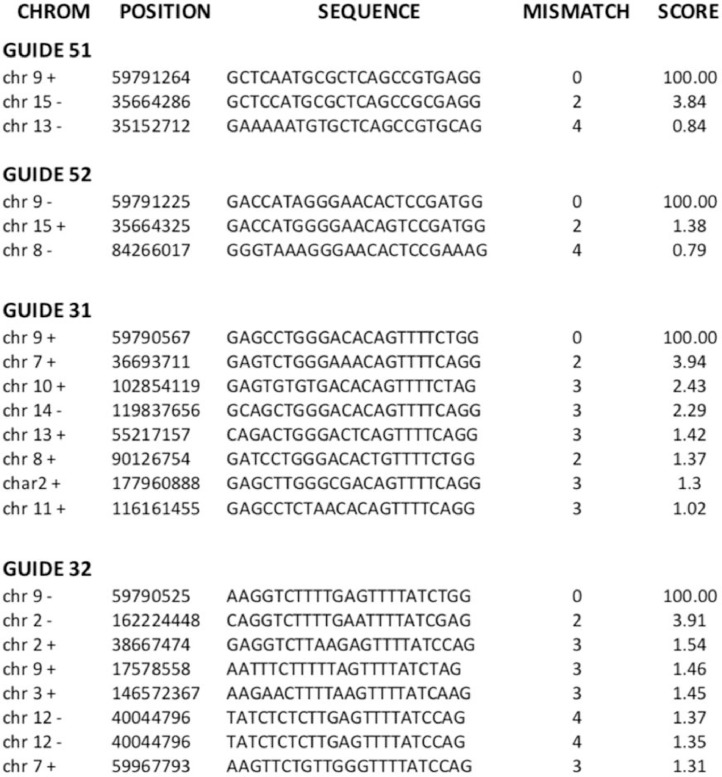
**List of on-target (100% match) and potential off-target regions (all showing very low scores for each guide) that were tested in all the pups.** No off-target events were detected.

From all the edited alleles, we selected alleles Δ27 (a short in-frame deletion at the 5′ target site) and ΔE8 (the designed medium size deletion of exon 8) for further analysis. By subsequent crosses, we generated heterozygous and homozygous strains of these two selected alleles, whose phenotypic and molecular characterization is described in the related research article [1]. In addition, potential off-target effects were analyzed using the Zhang Lab Tools for Guide Design (https://zlab.bio/guide-design-resources) (Fig. 5). Specific primers were designed and assayed in all the gene-edited animals, but no additional off-target site editing was detected.

## 2 Experimental Design, Materials and Methods

### 2.1 Generation of gene-edited mice using the CRISPR/Cas9 system

The CRIPSR/Cas9 system was used to generate a *Nr2e3* mouse model by deleting the exon 8 of the locus. To generate this *Nr2e3* mutant mice, zygotes from pregnant donor females were extracted, then modified and finally transferred to pseudo-pregnant receptor females (Fig. 1). Superovulation of donor females was achieved by administering 5IU PMSG (Pregnant Mare's Serum Gonadotropin) and, 47 h later, 5IU hCG (human Chorionic Gonadotropin) via intraperitoneal injection. Immediately after hGC administration, females were mated with males [18]. 24–48 h post-mating, several murine zygotes (in a one or two-cell state) from pregnant donor females were extracted [19] and microinjected with a number of guide-RNA and the endonuclease Cas9-D10A mRNA. To minimize potential off-targets, D10A Cas9, one of the nickase mutants of Cas9, was used. Four guides were designed, two guides per deletion site, in such a way to ensure single strand breaks in the targeted acceptor site of intron 7 and 3′ UTR region of *Nr2e3* locus (Fig. 2) and minimize potential off-target effects. A schematic representation of the gRNA location in the *Nr2e3* locus can be found in the related research article [1]). Microinjected zygotes were subsequently transferred to pseudo-pregnant receptor females [20, 21]. To induce the pseudo-pregnant state in the receptor females, they were crossed with vasectomized males, which presented an excision of the testis vas deferens [22] and were capable to mate but unable to fertilize the female. The copulation induced a pseudo-pregnancy state in the receptor females, optimal for the embryo implantation and gestation. All embryonic procedures up to the generation of the chimaera founder mice (Fig. 1) were performed at the Mouse Mutant Core Facility, Institute for Research in Biomedicine (Barcelona, Spain).

### 2.2 Characterization of the offspring modified alleles

The offspring obtained was genotyped to characterize the modified alleles as well as to detect off-targets, if any, generated by the system. PCR products were electrophoresed in a resolutive high concentration agarose gel (3% agarose, composed of 1.5% normal agarose and 1.5% low melting agarose) that allowed to resolve different size alleles without the need to perform T7 Endonuclease I Assay. When PCR bands for CRISPR-edited alleles were identified, the deletion was validated by Sanger sequencing. To confirm that the CRISPR-Cas9 technique did not introduce any off-target deletion/mutation, all the potential off-target sequences (up to three mismatches with sgRNA) determined by a prediction software (https://zlab.bio/guide-design-resources) were analyzed (Fig. 5). Subsequently, the mice bearing alleles of interest were selected and crossed to obtain murine heterozygous and homozygous lines for the different mutations [23]. Mutants with a partial or complete deletion (Fig. 3 and Fig. 4) of the exon 8 and 3′-UTR regions were selected.

### 2.3 Genotyping

Mouse genomic DNA was isolated from ear biopsies following overnight digestion at 55 °C in a lysis buffer with proteinase K. DNA amplification by PCR was used to genotype the mouse colony. Primer pairs were used to discern between *Nr2e3* Δ27 allele (*Nr2e3* intron 7 Fw and exon 8 Rv), ΔE8 allele (*Nr2e3* intron 7 FW and down Rv), and WT alleles (primer sequences are detailed in the related research article [1]). Founder mice bearing the selected alleles were subsequently mated for heterozygous F_1_ offspring. After genotyping, animals carrying the selected allele were further mated until obtaining an F_2_ or F_3_ homozygous lineage to perform phenotypic studies.

## Ethics Statment

Animal handling, euthanasia and surgical dissection was performed according to the ARVO statement for the use of animals in ophthalmic and vision research, following the guidelines for animal care of the University of Barcelona and with the approval of the Bioethics Committee of the University of Barcelona (File references FUE-2019–00965313, ID 2MDLDY4WZ).

## Declaration of Competing Interest

The authors declare that they have no conflict of interest.

## References

[1] Aísa-Marín I., López-Iniesta M.J., Milla S., Lillo J., Navarro G., de la Villa P., Marfany G. (2020). *Nr2e3* functional domain ablation by CRISPR-Cas9D10A identifies a new isoform and generates retinitis pigmentosa and enhanced S-cone syndrome models. Neurobiol. Dis..

[2] Kim D., Kim S., Kim S., Park J., Kim J.S. (2016). Genome-wide target specificities of CRISPR-Cas9 nucleases revealed by multiplex Digenome-seq. Genome Res..

[3] Wang X. (2015). Unbiased detection of off-target cleavage by CRISPR-Cas9 and TALENs using integrase-defective lentiviral vectors. Nat. Biotechnol..

[4] Tsai S.Q. (2015). GUIDE-seq enables genome-wide profiling of off-target cleavage by CRISPR-Cas nucleases. Nat. Biotechnol..

[5] Frock R.L. (2015). Genome-wide detection of DNA double-stranded breaks induced by engineered nucleases. Nat. Biotechnol..

[6] Wu X. (2014). Genome-wide binding of the CRISPR endonuclease Cas9 in mammalian cells. Nat. Biotechnol..

[7] Kuscu C., Arslan S., Singh R., Thorpe J., Adli M. (2014). Genome-wide analysis reveals characteristics of off-target sites bound by the Cas9 endonuclease. Nat. Biotechnol..

[8] Cho S.W. (2014). Analysis of off-target effects of CRISPR/Cas-derived RNA-guided endonucleases and nickases. Genome Res..

[9] Pattanayak V. (2013). High-throughput profiling of off-target DNA cleavage reveals RNA-programmed Cas9 nuclease specificity. Nat. Biotechnol..

[10] Hsu P.D. (2013). DNA targeting specificity of RNA-guided Cas9 nucleases. Nat. Biotechnol..

[11] Fu Y. (2013). High-frequency off-target mutagenesis induced by CRISPR-Cas nucleases in human cells. Nat. Biotechnol..

[12] Cradick T.J., Fine E.J., Antico C.J., Bao G. (2013). CRISPR/Cas9 systems targeting beta-globin and CCR5 genes have substantial offtarget activity. Nucl. Acids Res..

[13] Chiang T-W.W., le Sage C., Larrieu D., Demir M., Jackson S.P. (2016). CRISPR-Cas9(D10A) nickase-based genotypic and phenotypic screening to enhance genome editing. Sci. Rep..

[14] Gopalappa R., Bharathi S., Suresh R., Hyongbum H.K. (2018). Paired D10A Cas9 nickases are sometimes more efficient than individual nucleases for gene disruption. Nucl. Acids Res..

[15] Ran F.A. (2013). Double nicking by RNA-guided CRISPR Cas9 for enhanced genome editing specificity. Cell.

[16] Cho S.W., Kim S., Kim Y., Kweon J., Kim H.S., Bae S., Kim J.S. (2014). Analysis of off-target effects of CRISPR/Cas-derived RNA-guided endonucleases and nickases. Genome Res..

[17] Guilinger J.P., Thompson D.B., Liu D.R. (2014). Fusion of catalytically inactive Cas9 to FokI nuclease improves the specificity of genome modification. Nat. Biotechnol..

[18] A mouse colony for production of transgenic and chimeric animals (Chapter 3, Embryo donors) in Nagy, A., et al. (Eds.), Manipulating the Mouse Embryo 4th ed. p. 89. Cold Spring Harbor Laboratory Press: Cold Spring Harbor, New York

[19] Opening the abdominal cavity and locating female reproductive organs (Chapter 4, Protocol 5) in Nagy, A., et al. (Eds.), Manipulating the Mouse Embryo 4th ed. p. 137. Cold Spring Harbor Laboratory Press: Cold Spring Harbor, New York.

[20] Oviduct transfer of mouse embryos (Chapter 6, Protocol 3) in Nagy, A., et al. (Eds.), Manipulating the Mouse Embryo 4th ed. p. 211. Cold Spring Harbor Laboratory Press: Cold Spring Harbor, New York.

[21] Cesarean section and fostering of mice (Chapter 6, Protocol 5) in Nagy, A., et al. (Eds.), Manipulating the Mouse Embryo 4th ed. p. 220. Cold Spring Harbor Laboratory Press: Cold Spring Harbor, New York.

[22] Vasectomy of mice (Method 2: scrotal) in Nagy, A., et al. (Eds.), Manipulating the Mouse Embryo 4th ed. p. 210. Cold Spring Harbor Laboratory Press: Cold Spring Harbor, New York.

[23] Production of Chimeras (Chapter 12) in Nagy, A., et al. (Eds.), Manipulating the Mouse Embryo 4th ed. p. 489. Cold Spring Harbor Laboratory Press: Cold Spring Harbor, New York.

